# Self-powered flexible all-perovskite X-ray detectors with high sensitivity and fast response

**DOI:** 10.1016/j.isci.2021.102927

**Published:** 2021-07-30

**Authors:** Jin Hyuck Heo, Jin Kyoung Park, Yang (Michael) Yang, David Sunghwan Lee, Sang Hyuk Im

**Affiliations:** 1 Department of Chemical and Biological Engineering, Korea University, 145 Anam-ro, Seongbuk-gu, Seoul 136-713, Republic of Korea; 2 State Key Laboratory of Modern Optical Instrumentation, College of Optical Science and Engineering, International Research Center for Advanced Photonics, Zhejiang University, Hangzhou, Zhejiang, China

**Keywords:** Physics, Engineering, Devices

## Abstract

Perovskite materials have demonstrated superior performance in many aspects of optoelectronic applications including X-ray scintillation, photovoltaic, photodetection, and so on. In this work, we demonstrate a self-powered flexible all-perovskite X-ray detector with high sensitivity and fast response, which can be realized by integrating CsPbBr_3_ perovskite nanocrystals (PNCs) as the X-ray scintillator with a CH_3_NH_3_PbI_3_ perovskite photodetector. The PNCs scintillator exhibits ultra-fast light decay of 2.81 ns, while the perovskite photodetector gives a fast response time of ∼0.3 μs and high-specific detectivity of ∼2.4×10^12^ Jones. The synergistic effect of these two components ultimately leads to a self-powered flexible all-perovskite X-ray detector that delivers high sensitivity of 600–1,270 μC/mGy_air_cm^3^ under X-ray irradiation and fast radiation-to-current response time.

## Highlights

• Self-powered flexible all-perovskite indirect X-ray detectors are fabricated
• CsPbBr_3_ PNCs-PDMS composite X-ray scintillator shows excellent super flexibility
• The MAPbI_3_ photodetector has good response to both visible and X-ray irradiation
• The X-ray detector shows high sensitivity, fast response, and good bending stability

## Graphical abstract

**Figure:**
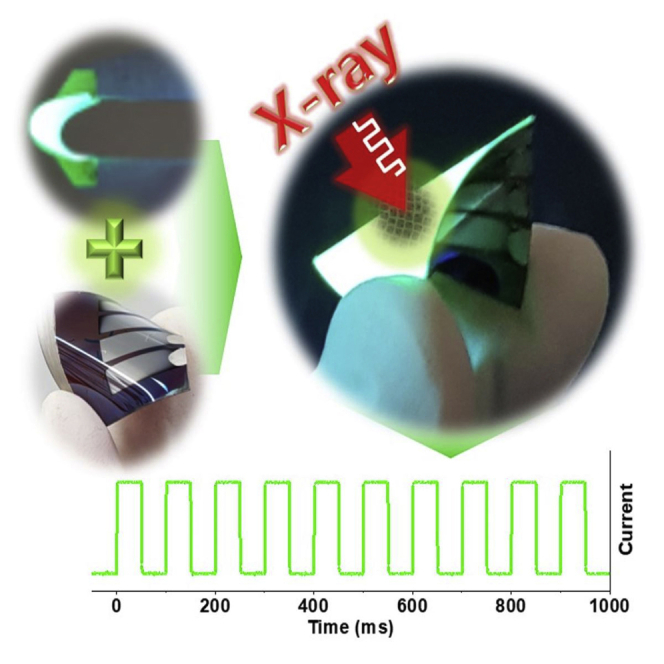

## Introduction

Since Wilhelm Röntgen discovered X-rays, extensive studies have been done to find their commercial applications such as crystallography, medical inspection and therapy, non-destructive industrial inspection, security check, and space exploration (Blasse, 1994; Tegze and Faigel, 1996; Rieder et al., 1997). The X-ray has been generally used to obtain the X-ray diffraction patterns of crystals, mammography, intra-oral structure, computed tomography (CT), and airport security scan. The emission spectrum of an X-ray source contains a characteristic X-ray with spike shape and Bremsstrahlung X-ray with broad spectrum, where the characteristic X-ray with ∼30 keV energy is used for mammography and the broad Bremsstrahlung is exploited for chest radiography. The allowed X-ray spectrum to be used for detection also depends on the part of human body for diagnosis because the damage by X-ray irradiation varies with organs such as tissue and bone. Therefore, it is desirable for the X-ray detector to have good performance at a broad X-ray spectrum region for wide application.

The X-ray detectors can be classified as direct and indirect types. The former directly captures the X-ray photoelectrons generated in an X-ray absorption layer such as amorphous Se (a-Se) under applied bias voltage, so it can obtain high resolution. The direct X-ray detector is currently used in relatively soft X-ray detection and imaging such as mammography. The detection of hard X-ray is performed mostly through an indirect scenario (Su et al., 2020). It relies on a scintillator such as thallium doped cesium iodide (CsI:Tl) (Grassmann et al., 1985) and terbium-doped gadolinium oxysulfide (Gd_2_O_2_S:Tb, GOS) (Van Eijk, 2002), to convert X-ray to visible photons, which can be further detected by photodetectors such as Si photodiodes. The indirect type X-ray detectors have captivated most markets today because they are cheaper and more stable than the direct type detectors. However, it is still a great challenge to develop indirect type X-ray detectors with high sensitivity, high resolution, and a fast scan rate in order to minimize radiation exposure to the patient.

In 2015, Yakunin et al. first reported on a direct type CH_3_NH_3_PbI_3_ (MAPbI_3_) perovskite X-ray detector with high sensitivity (25 μC/mGy_air_cm^3^) and responsivity (1.9×10^4^ carriers/photon) (Yakunin et al., 2015). Wei et al. then fabricated a direct type MAPbBr_3_ single crystal perovskite X-ray detector with an improved sensitivity of to 80 μC/mGy_air_cm^2^, which is four folds higher than the sensitivity of a-Se detector (Wei et al., 2016). In 2017, a large-area (50 × 50 cm^2^) MAPbX_3_ (X = Cl, Br or I) perovskite X-ray direct-type detector was fabricated by Kim et al. through a printing process (Kim et al., 2017). The large area device exhibited a sensitivity of 11 μC/mGy_air_cm^2^ and they were able to obtain X-ray images from it. Other than the MAPbI_3_ perovskite, CsPbBr_3_ based perovskite was also soon realized as an excellent candidate for X-ray detection particularly due to its high Z value coming from the heavy Cs atoms. Pan et al. fabricated direct-type CsPbBr_3_ X-ray detectors by employing a hot-pressing method to form a ∼240 μm thick quasi-monocrystalline CsPbBr_3_ film, and reported on an excellent sensitivity of 55.684 μC/mGy_air_cm^2^ (Pan et al., 2019). However, the direct-type perovskite X-ray detectors have been fabricated by depositing very thick crystalline perovskite layer on thin film transistor (TFT) arrays or complementary metal oxide semiconductor (CMOS) arrays, so it is difficult to make flexible X-ray detectors due to the brittle thick perovskite layer.

In contrast, Heo et al. reported an indirect type scintillator-based X-ray detector consisting of CsPbBr_3_ perovskite nanocrystals (PNCs), which showed better spatial resolution of 9.8 lp/mm at a modulation transfer function (MTF) of 0.2 and 12.5 lp/mm for a linear line chart, compared to the GOS scintillator-based detector (6.2 lp/mm at MTF = 0.2 and 6.3 lp/mm for a linear line chart) (Heo et al., 2018). The CsPbBr_3_ PNCs were dispersed in a relatively rigid poly-methylmethacrylate (PMMA) matrix as the scintillator and the arrayed photodetectors were formed on a rigid substrate, thereby preventing the indirect type X-ray detector from exhibiting flexibility.

Development of portable flexible X-ray detectors with high sensitivity and fast response is very useful for analyzing a structure of curved architecture and to distinguish materials such as soft and hard matters. Currently, the portable chest X-ray has contributed toward detecting lungs infected by the coronavirus disease-19 (COVID-19) (Jacobi et al., 2020). Accordingly, it is important to fabricate a flexible scintillator that can be self-powered in order to develop portable X-ray detectors with high performance. So far, a wealth of studies on flexible perovskite solar cells (Heo et al., 2019; Jung et al., 2019) and photodetectors (Wang and Kim, 2017) have been done, and recently Gill et al. reported that a MAPbI_2_Cl perovskite is ∼550% more sensitive for X-ray detection than the commonly used a-Si devices (Gill et al., 2018). Hence, here we fabricated a flexible CsPbBr_3_ PNCs-based scintillator combined with a self-powered flexible MAPbI_3_ perovskite photodetector without requiring external bias voltage. By combining the flexible PNCs-based scintillator and the flexible perovskite photodetector, we could demonstrate all-perovskite flexible self-powered X-ray detectors with high sensitivity and fast response.

## Results and discussion

### Fabrication and X-Ray scintillation of flexible CsPbBr_3_ PNCs film

**Figure 1:**
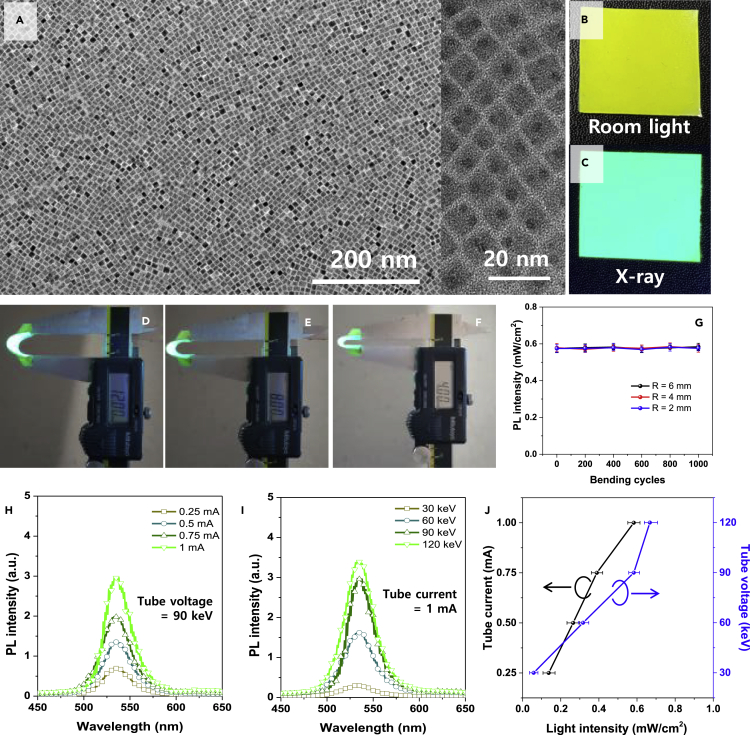
CsPbBr_3_ perovskite nanocrystals (PNCs) X-ray scintillator (A) Transmission electron microscopy (TEM) image of CsPbBr_3_ PNCs: inset TEM image = magnified image. Scale bar of figure is 200 nm, and scale bar of inset is 20 nm. (B and C) Photographs of CsPbBr_3_ PNCs scintillator film under (B) room light and (C) X-ray irradiation. (D–G) Photographs of bent PNCs scintillator films emitting PL under irradiation of X-ray (tube voltage = 90 keV, tube current = 1 mA) with bending radii of (D) 6 mm, (E) 4 mm, and (F) 2 mm, and (G) bending stabilities of the PNCs scintillator films showing variations of PL intensities with repeated bending cycles. (H–J) PL spectra of the PNCs scintillator film with (H) various tube current at 90 keV tube voltage and (I) various tube voltage at 1 mA tube current, and (J) light intensity emitted from the PNCs scintillator film corresponding to (H) and (I). Data with error bars are represented as mean +/− standard deviation.

Figure 1A is a transmission electron microscopy (TEM) image of CsPbBr_3_ PNCs, which were synthesized by previously reported procedures involving solution chemistry (Heo et al., 2018). The synthesized CsPbBr_3_ PNCs had ∼10 nm-sized nanocubes or nanobars and were uniformly dispersed. The inset TEM image indicates that the CsPbBr_3_ PNCs have a cubic crystal structure exposing the {100} facet. Further characterizations of CsPbBr_3_ PNCs such as X-ray diffraction (XRD) pattern, UV-visible absorption spectrum, static photoluminescent (PL) spectrum, and dynamic transient PL spectrum are shown in Figure S1. The XRD pattern in Figure S1A confirms that the synthesized CsPbBr_3_ PNCs have a cubic phase, which is consistent with the TEM results. The UV-visible absorption and static PL spectrum in Figure S1B indicate that the CsPbBr_3_ PNCs have an on-set absorption band edge at a wavelength of ∼510 nm and a strong single PL peak at a wavelength of ∼520 nm-wavelength with a full width at half maximum (FWHM) value of ∼20 nm. The dynamic transient PL spectrum in Figure S1C indicates that the CsPbBr_3_ PNCs have an average PL life-time of 2.81 ns (τ_1_ = 0.42 ns (48.77%), τ_2_ = 5.16 ns (51.23%)).

Figures 1B and 1C are photographs of the flexible CsPbBr_3_ PNCs scintillator film, which was made by mixing the CsPbBr_3_ PNCs with poly-dimethylsiloxane (PDMS) resin and a curing agent, subsequently followed by the degassing and curing processes at 60°C for 12 hr at N_2_ atmosphere, under room light (Figure 1B) and X-ray (90 keV, 1 mA) irradiation (Figure 1C). The strong blue-green PL emission of the PNCs scintillator film implies that the CsPbBr_3_ PNCs can emit PL under a low X-ray dose. The average PLQY (PL quantum yield) of the scintillator film was 63.5 ± 3.57% as shown in Figure S1D.

Figures 1D–1F are photographs of the flexible PNCs scintillators bent at bending radii (r) of r = 6 mm (Figure 1D), r = 4 mm (Figure 1E), and r = 2 mm (Figure 1F) under X-ray exposure (tube voltage = 90 keV, tube current = 1 mA). It should be noted that here r is the outer bending radius. If we consider the thickness of the PNCs scintillator film (thickness = 1.5 mm), the inner bending radius is much smaller than the outer r. Figure 1G shows bending stabilities of the PNCs scintillator films. All samples maintained their PL intensities irrespective of the repeated bending cycles up to 1,000 cycles. Therefore, the CsPbBr_3_ PNCs scintillator film has excellent bending stability and sufficient flexibility because its matrix is made by super flexible PDMS rubber.

Figures 1H and 1I are the PL intensity dependence of the PNCs scintillator film under a fixed tube voltage of 90 keV (Figure 1H) and under a fixed tube current of 1 mA (Figure 1I). Both PL spectra show a strong emission of green light at a wavelength of ∼533 nm while their intensities gradually increased as either the tube current or tube voltage increased. Evidently, the PL intensities are strongly dependent on the dose rate of the irradiated X-rays, implying that the flexible PNCs scintillator film can respond to a broad X-ray photon energy spectrum while exhibiting a linear response with X-ray dose rate. To exactly measure the correlation of tube current vs PL intensity and tube voltage vs PL intensity, we measured again the PL intensities with a photodetector as shown in Figure 1J, because the indirect type X-ray detector would also measure the PL intensity originating from the scintillator film.

### Self-powered flexible MAPbI_3_ perovskite photodetector with fast response

**Figure 2:**
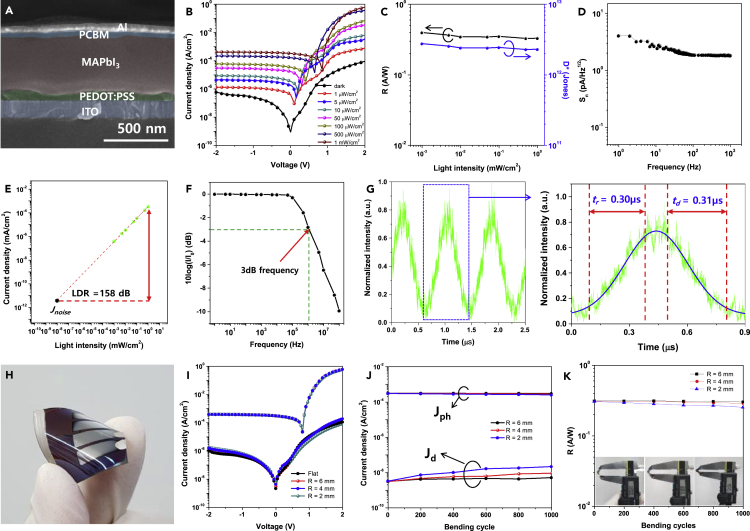
Self-powered MAPbI_3_ perovskite photodetector (A) Scanning electron microscopy (SEM) cross-sectional image of rigid MAPbI_3_ perovskite photodetector composed of glass/indium tin oxide (ITO)/poly(3,4-ethylenedioxythiophene):poly(styrenesulfonate) (PEDOT:PSS)/MAPbI_3_/[6,6]-phenyl-C61-butyric acid methyl ester (PCBM)/Al. Scale bar is 500 nm. (B–G) (B) Current density-voltage (J-V) curves under various light intensities, (C) responsivity (R) and specific detectivity (D∗) dependence on various light intensity, (D) noise spectral density (S_n_) as a function of frequency, (E) linear dynamic range (LDR), (F) signal attenuation with respect to frequency, and (G) output photocurrent signal under 1 MHz input pulse modulation of the perovskite photodetector. (H–K) (H) Photograph of the flexible perovskite photodetector with a poly(ethylene terephthalate) (PET) substrate, (I) J-V curves of the perovskite photodetector bent at bending radii of ∞ (flat), 6, 4, and 2 mm, (J) bending stability of the perovskite photodetector with repeated bending cycles, and (K) the responsivity of perovskite photodetector with repeated bending cycles with photographs of the bent flexible devices in the inset.

Figure 2A is a scanning electron microscopy (SEM) cross-sectional image of the self-powered MAPbI_3_ perovskite photodetector, which is composed of glass/indium tin oxide (ITO)/poly(3,4-ethylenedioxythiophene):poly(styrenesulfonate) (PEDOT:PSS)/MAPbI_3_/[6,6]-phenyl-C61-butyric acid methyl ester (PCBM)/Al. The thickness of each layer was ∼150/∼50/∼400/∼50/∼50 nm for ITO/PEDOT:PSS/MAPbI_3_/PCBM/Al, respectively.

The current density-voltage (J-V) curves of the perovskite photodetector under various light intensities are shown in Figure 2B. The light intensity was controlled from 1 μW/cm^2^ to 1 mW/cm^2^ with a 510 nm-wavelength laser. The J-V curves clearly show that the current density at zero bias potential gradually increases with increasing intensity of the irradiated light. The responsivity (R) and specific detectivity (D∗) can be written as follows:

(Equation 1) R = (J_ph_ − J_d_) P^−1^ [A/W]

where J_ph_ is the photocurrent density, J_d_ is the dark current density, and P is the incident light intensity, and:

(Equation 2) D∗ = RA^0.5^S_n_^−1^ [cmHz^0.5^/W = Jones]

where R, A, and S_n_ stand for responsivity [A/W], photosensitive area [cm^2^], and noise spectral density [A/Hz^0.5^], respectively.

The calculated R and D∗ values relative to light intensity for the self-powered MAPbI_3_ perovskite photodetector were plotted in Figure 2C, as J_ph_, J_d_ and P are obtainable in Figure 2B, and S_n_ is ∼4.0 × 10^−12^ A/Hz^0.5^ from Figure 2D. The calculated R and D∗ of the self-powered detector were ∼0.35 A/W and ∼2.4×10^12^ Jones, respectively. The log(current density)-log(light intensity) plot in Figure 2E indicates that the linear dynamic range (LDR) of the perovskite photodetector is ∼158 dB. The signal attenuation (10log(I×I_0_^−1^)) with frequency was shown in Figure 2F, which indicates that the 3 dB penalty frequency of the perovskite photodetector is ∼5 MHz. This implies that the self-powered MAPbI_3_ perovskite photodetector can process information up to the MHz frequency range. To confirm this, we acquired a 1 MHz signal through the perovskite photodetector as shown in Figure 2G. The magnified signal show that the rising time (τ_r_) and decay time (τ_d_) of the response signals of perovskite photodetector are 0.30 μs and 0.31 μs, respectively. Accordingly, the perovskite photodetector can effectively acquire real-time information.

To fabricate a flexible self-powered X-ray detector, both the PNCs scintillator and perovskite photodetector must have sufficient mechanical flexibility and bending stability. The mechanical flexibility and stability of the PNCs scintillator were confirmed in the previous section. Similarly, the mechanical flexibility and stability of the perovskite photodetector are shown in Figures 2H–2K. A photograph of a flexible perovskite photodetector (size = 2.54 × 2.54 cm^2^, each active area = 0.16 cm^2^) composed of poly(ethylene terephthalate) (PET)/ITO/PEDOT:PSS/MAPbI_3_/PCBM/Al is shown in Figure 2H. The J-V curves of the flexible perovskite photodetector bent to specific bending radii (r = ∞ (flat), 6, 4, and 2 mm) under dark and photo conditions (1 mW/cm^2^) are shown in Figure 2I. Apparently, the J-V curves indicate that the J_ph_ and J_d_ of the bent photodetector are almost constant irrespective of bending radius due to its small active area. This implies that the flexible perovskite photodetector can acquire certain information signals without significant distortion under bent circumstances. To check the bending stability of the perovskite detector, the J_ph_ and J_d_ of the flexible perovskite photodetector were measured with respect to the repeated bending cycles as shown in Figure 2J. Interestingly, the J_ph_ of the repeatedly bent perovskite photodetector was almost constant regardless of the repeated bending cycles, while J_d_ was slightly degraded with repeated bending cycles. The degradation amount of J_d_ was also slightly increased as the bending radius decreased. The responsivities of the flexible perovskite photodetectors with respect to the repeated bending cycles are shown in Figure 2K, and the inset shows photographs of flexible perovskite photodetectors bent to r = 6, 4, and 2 mm. Since R is a function of (J_ph_ − J_d_)P^−1^, the responsivities of the flexible perovskite photodetector were almost constant up to a repeated bending test of 1,000 cycles but very slightly deteriorated with decreasing bending radius due to the increasing J_d_.

### Device performance of self-powered all-perovskite X-Ray detectors

**Figure 3:**
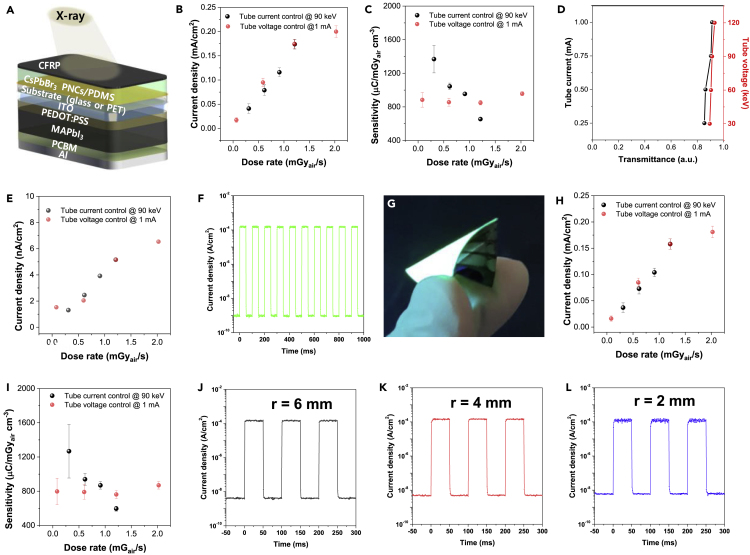
Self-powered all-perovskite X-ray detector (A–F) (A) Schematic device structure of the self-powered all-perovskite X-ray photodetector, (B) current densities of rigid glass substrate-based device as a function of X-ray dose rate, (C) the corresponding X-ray sensitivities with dose rate, (D) X-ray transmittance of the MAPbI_3_ perovskite photodetector with various tube current at 90 keV tube voltage and various tube voltage at 1 mA tube current, (E) the corresponding current densities of perovskite photodetector with dose rate, and (F) output signals of the self-powered all-perovskite X-ray detector under in response to rectangular input X-ray signals with a 50 ms time interval. (G–L) (G) Photograph of the flexible substrate-based self-powered all-perovskite X-ray detector, (H) current densities with respect to dose rate, (I) the corresponding X-ray sensitivities with dose rate, and output signals of the flexible X-ray detector with bending radii of (J) 6, (K) 4, and (L) 2 mm under exposure of rectangular input X-ray signals with a 50 ms time interval. Data with error bars are represented as mean +/− standard deviation.

Figure 3A is the schematic device structure of the self-powered all-perovskite X-ray detector composed of carbon fiber reinforced polymer (CFRP) film/CsPbBr_3_ PNCs scintillator film/substrate (glass for rigid device or PET for flexible device)/ITO/PEDOT:PSS/MAPbI_3_ perovskite/PCBM/Al. Figure 3B shows the current densities detected by the photodetector with respect to the X-ray dose rate. The current density of perovskite photodetector linearly increases with increasing X-ray dose rate. Under a fixed tube current of 1 mA, the all-perovskite X-ray detector acquired current density values of 0.017–0.199 mA/cm^2^ as the tube voltage increased from 30 to 120 keV, whereas under a fixed tube voltage of 90 keV, the detector current density values ranged from 0.041 to 0.174 mA/cm^2^ as the tube current increased from 0.25 to 1 mA.

The X-ray sensitivity (S) can be expressed as follows (Thirimanne et al., 2018):

(Equation 3) S = [∫(J_x-ray_(t) – J_dark_)dt](DA_t_)^−1^

where J_x-ray_(t), J_dark_, D, and A_t_ stand for current density generated by X-ray irradiation during time (t), dark current density without X-ray irradiation, X-ray dose, and thickness of active region.

When calculating S in an active area basis, the thickness term represented by A_t_ can be neglected. Here, we fixed the active area, the thickness of PNCs scintillator, and the thickness of the perovskite photodetector to 0.16 cm^2^, 0.15 cm, and 4 × 10^−5^ cm, respectively. Overall, the self-powered all-perovskite X-ray detector exhibited sensitivity values of 130–140 μC/mGy_air_cm^2^ in an active area basis and 880 to 960 μC/mGy_air_cm^3^ in an active volume basis, when the tube voltage was fixed to 90 keV, compared with the sensitivity values ranging from 100 to 210 μC/mGy_air_cm^2^ in an active area basis and from 660 to 1,370 μC/mGy_air_cm^3^ in an active volume basis, when the tube current was otherwise fixed at 1 mA, as shown in Figure 3C. The correlations between transmittance and dose rate of PNCs scintillator are shown in Figure S3. The constant sensitivity under a fixed tube voltage irrespective of the variation in tube current is attributed to the constant absorptivity of the CsPbBr_3_ PNCs scintillator as shown in Figure 1, Figure 2, Figure 3B . In contrast, the decreasing sensitivity under fixed tube current with increasing tube voltage is caused by the decreasing absorptivity of PNCs scintillator with dose rate as shown in Figure S3C.

The ∼400-nm-thick MAPbI_3_ perovskite photodetector itself is also responsible for X-ray detection, behaving similarly to a direct type X-ray detector. The X-ray absorbance of the perovskite photodetector was below 10% as shown in Figure 3D, thus generating a current density of 1–7 nA/cm^2^ as shown in Figure 3E. Considering that the net generated current density by the all-perovskite X-ray detector under the same X-ray irradiation ranged from 0.017 to 0.174 mA/cm^2^, the contribution of the perovskite photodetector to the detection signal can be neglected. Figure 3F shows the response time of the self-powered all-perovskite X-ray detector, indicating that the output signals exhibit no time delay in response to the rectangular input X-ray signal pulses with a 50 ms time interval, irradiating upon the detector. This fast response of the X-ray detector can be attributed to a very short PL life-time of the CsPbBr_3_ PNCs scintillator film (2.81 ns) and quick response time of MAPbI_3_ perovskite photodetector (∼0.3 μs).

Finally, we fabricated a flexible self-powered all-perovskite X-ray detector composed of CsPbBr_3_ PNCs scintillator/PET/ITO/PEDOT:PSS/MAPbI_3_/PCBM/Al and its photograph was shown in Figure 3G. The current densities generated by the X-ray irradiation are shown in Figure 3H, which exhibits similar results to the rigid glass based all-perovskite X-ray detector. Additionally, the X-ray sensitivity dependences on the dose rate for the flexible X-ray detector are shown in Figure 3I. The X-ray sensitivity ranged from 120 to 130 μC/mGy_air_cm^2^ in an active area basis and from 800 to 870 μC/mGy_air_cm^3^ in an active volume basis under a fixed tube voltage of 90 keV, and ranged from 90 to 190 μC/mGy_air_cm^2^ in an active area basis and from 600 to 1,270 μC/mGy_air_cm^3^ in an active volume basis under a fixed tube current of 1 mA. It is important to check if the X-ray signals of the flexible all-perovskite X-ray detector were distorted during the bending stages, so we acquired output signals of the flexible all-perovskite X-ray detector at bending radii of r = 6 (Figures 3J), 4 (Figure 3K), and 2 mm (Figure 3L), respectively, when under irradiation of rectangular input X-ray pulse signals with 50 ms time interval. It can be seen that the flexible self-powered perovskite X-ray detector did not exhibit signal distortion, which is a combined result of both the flexible CsPbBr_3_ PNCs scintillator (Figure 1G) and the flexible MAPbI_3_ perovskite photodetector (Figures 2I–2K) not showing significant signal distortion under the same bending conditions (r = 6, 4, and 2 mm). This implies that the flexible detector can acquire X-ray images without distortion when attached to curved or bent objects. A comparison chart of sensitivities from this work and among various X-ray detectors from other works can be seen in Figure S4 and Table S1.

### Conclusion

In summary, we fabricated self-powered flexible all-perovskite X-ray detectors with high sensitivity and fast response time. The flexible X-ray detector was fabricated by combining a CsPbBr_3_ PNCs scintillator and an MAPbI_3_ perovskite photodetector in order to take advantage of both indirect and direct-type X-ray detectors. The flexible PNCs X-ray scintillator was made by dispersing PNCs in PDMS matrix, and exhibited super-flexibility and quick response (PL life-time = 2.81 ns). The PNCs scintillator efficiently emitted PL under X-ray irradiation with a broad X-ray energy spectrum (30-120 keV) and low dose. Meanwhile, the MAPbI_3_ perovskite photodetector exhibited photodetection parameters of R = ∼0.35 A/W, D∗ = ∼ 2.4×10^12^ Jones, LDR = ∼158 dB, and a 3 dB signal response frequency of ∼5 MHz (response time = ∼0.3 μs). Accordingly, the self-powered flexible all-perovskite X-ray detector that integrates a PNCs scintillator and a perovskite photodetector showed a high sensitivity range of 600–1,270 μC/mGy_air_cm^3^ under X-ray irradiation (tube current = 1 mA, and tube voltage 30-120 keV) without the need of applying bias voltage and a very quick response time. In addition, the all-perovskite X-ray detector maintained its performance under severe bending conditions (r = 6, 4, and 2 mm) and even after a rigorous repeated bending test of 1,000 cycles. We believe that the flexible all-perovskite X-ray detector holds potential as a versatile X-ray detector which can be applicable to broad applications such as mammography, CT, medical inspection, security check, and industrial inspection, largely attributed to its high sensitivity, high resolution, fast response time, and versatility by simply switching among different PNCs scintillators suitable for specific applications.

### Limitations of the study

For the X-ray experiments, a specialized lead chamber and the supervision of an authorized specialist with a certification of X-ray source handling are necessary. Please see the caution in STAR Methods section.

## STAR★Methods

### Key resources table

**Table:** 

REAGENT or RESOURCE	SOURCE	IDENTIFIER
**Chemicals, peptides, and recombinant proteins**		

Cesium carbonate (Cs_2_CO_3_)	Sigma-Aldrich	202126; CAS: 534-17-8
Oleic acid (OA)	Sigma-Aldrich	364525; CAS: 112-80-1
Octadecene (ODE)	Sigma-Aldrich	O806; CAS:112-88-9
Lead (II) Bromide (PbBr_2_)	Sigma-Aldrich	398853; CAS: 10031-22-8
Oleylamine (OLA)	ACROS Organics	AC129540050; CAS: 112-90-3
Hexane	Sigma-Aldrich	296090; CAS: 110-54-3
PDMS monomer (Sylgard 184A)	Sewang Hitech	
Curing agent (Sylgard 184B)	Sewang Hitech	
Poly(3,4-ethylenedioxythiophene):poly(styrenesulfonic acid) (PEDOT:PSS)	Clevios	AI4083
Methanol	Merk	106009; CAS: 67-56-1
Methylammonium iodide (MAI)	Greatcell Solar	MS101000; CAS: 14965-49-2
Lead (II) iodide (PbI_2_)	Sigma-Aldrich	211168; CAS: 10101-63-0
N,N-dimethylformamide (DMF)	Sigma-Aldrich	227056; CAS: 68-12-2
Hydriodic acid (HI)	Sigma-Aldrich	210021; CAS: 10034-85-2
Phenyl-C61-butyric acid methyl ester (PCBM)	Nano-C	CAS: 160848-22-6
Toluene	Sigma-Aldrich	244511; CAS: 108-88-3

**Software and algorithms**		

Origin 2018	https://www.originlab.com	N/A

### Resource availability

#### Lead contact

Further information and requests for resources and materials should be directed to and will be fulfilled by the lead contact, Sang Hyuk Im (imromy@korea.ac.kr).

#### Materials availability

This study did not generate new unique reagents.

### Method details

#### Preparation of CsPbBr_3_ PNCs

To synthesize the CsPbBr_3_ perovskite NCs, we prepared the Cs-oleate solution by reacting 0.814 g of Cs_2_CO_3_ (Aldrich, 99.9%) with 2.5 mL of oleic acid (OA, Aldrich 90%) in 40 mL of octadecene (ODE, Aldrich, 90%) at 150°C under N_2_ condition until all Cs_2_CO_3_ reacted with OA. Afterward, we prepared the PbBr_2_ precursor solution by reacting 0.069 g of PbBr_2_ (99.999%, Aldrich) with 0.5 mL of oleylamine (OLA, Acros, 80-90%) and 0.5 mL of OA in 5 mL of ODE at 150°C under N_2_ condition for 1h. After preparing the both solution, 0.4 mL of Cs-oleate solution was rapidly injected into the prepared PbBr_2_ precursor solution. The reaction mixture was reacted at 150°C for 10 s and then cooled by ice-water bath. After cooling down, the CsPbBr_3_ PNCs were separated from solvent by centrifugation, and then re-dispersed in hexane (Aldrich, anhydrous 95%).

#### Fabrication of CsPbBr_3_ PNCs X-Ray scintillator

For fabrication of CsPbBr_3_ PNCs X-ray scintillator, we mixed in a 10:1 weight ratio of PDMS monomer (SYLGARD 184A, SEWANG HITECH CO. LTD.) and curing agent (SYLGARD 184B, SEWANG HITECH CO. LTD.). After then, 1 mL of CsPbBr_3_ PNCs solution (concentration of CsPbBr_3_ PNCs of ca. 0.5g/mL) was added to PDMS monomer/curing agent mixture and mixed. The mixture was poured into the container (3 cm × 3 cm × 1.7 mm) and then the excess mixture was removed using a bar. The mixture was transferred to a vacuum oven and degassed for 1 h to remove bubbles and solvent. After degassing process, the polymerization took place at 60°C for 12 h. Finally, CsPbBr_3_ PNCs X-ray scintillator was obtained by peeling off the polymerized film from container. The final size of CsPbBr_3_ PNCs X-ray scintillator is 3 cm × 3 cm × 1.5 mm due to slight shrinkage during polymerization.

#### Fabrication of MAPbI_3_ perovskite photodetector

To fabricate the MAPbI_3_ perovskite photodetector, we firstly spin-coated filtered poly(3,4-ethylenedioxythiophene):poly(styrenesulfonic acid) (PEDOT:PSS, Clevios, Al4083)/methanol (1:2 vol.:vol.) on a cleaned indium-doped tin oxide (ITO) glass substrate at 3000 rpm for 60 s and dried at 150°C for 20 min. A 40 wt% MAPbI_3_/DMF (N,N-dimethylformamide, Aldrich, 99%) solution with hydriodic acid additive (40 wt% MAPbI_3_ in DMF solution/hydriodic acid = 1 mL/100 μL) was then spin coated on the PEDOT:PSS/ITO substrate at 3000 rpm for 200 s and was dried on a hot plate at 100°C for 2 min. A (PCBM, nano-C) layer was deposited on the MAPbI_3_/PEDOT:PSS/ITO substrate by spin-coating PCBM/toluene (20 mg/mL) solution at 2000 rpm for 60 s. Finally, Al counter electrode was deposited by thermal evaporation.

#### Characterization of MAPbI_3_ perovskite photodetector

The current density-voltage (J-V) curves were measured potentiostat (IVIUM, IviumStat). DC noise was measured with a dynamic signal analyzer (Agilent 35670A) connected to a low noise current preamplifier (Stanford Research SR570) in the frequency range of 1 Hz to 1 kHz. The devices were illuminated by Xenon light source (ABET, 150 W Xenon lamp, 13014) with a monochromator (DONGWOO OPTRON Co., Ltd., MonoRA-500i). The light intensity was controlled by varying the current with neutral density filter (Thorlabs). The incident light intensity was calibrated by power meter (Newport, Model 1936-R). The pulse response measurements of devices were measured by 510 nm pulsed laser diode (HAMAMATSU, PLP-10).

#### Charaterization of CsPbBr_3_ PNCs X-ray scintillator and all-perovskite X-ray detector

The X-ray intensities were measured with X-ray dose meter (Magicmax Rad, Daol). The PL spectra of CsPbBr_3_ PNCs X-ray scintillator were measured by optical fiber connected to spectrometer (Ocean Optics, HR2000+). The light intensity emitted from CsPbBr_3_ PNCs X-ray scintillator were measured by power meter (Newport, Model 1936-R). TRPL spectra of CsPbBr_3_ PNCs X-ray scintillator was obtained by TRPL measurement system (PC1, ISS) with 373 nm pulsed laser.ort, Model 1936-R). TRPL spectra of CsPbBr_3_ PNCs X-ray scintillator was obtained by TRPL measurement system (PC1, ISS) with 373 nm pulsed laser. The PLQY of the CsPbBr_3_ perovskite scintillator film was measured using commercial PLQY spectrometer (Absolute PL quantum yield spectrometer: C9920-02, Hamamatsu) with an optical detector (PMA-12: C10027-01, Hamamatsu). The excitation light source was a 150 W Xenon lamp and the excitation wavelength was a 350 nm adjusted with a monochromator. The current density of all-perovskite perovskite X-ray detector under X-ray irradiation was measured by potentiostat (IVIUM, IviumStat). The X-ray response time of all-perovskite X-ray detector was measured with an X-ray source with a pulse width of 50 ms formed using a lead-covered mechanical chopper and current values were recorded by oscilloscope (MSO46, Tektronix).

##### Caution

All experiments involving the usage of X-ray were conducted within a specialized lead chamber under the supervision of a specialist authorized in handling an X-ray source. The chamber consists of two rooms separated by a lead door, where one room contains the X-ray source, and the other room is designated for remotely controlling the X-ray source. In order to perform X-ray characterizations, the sample was first placed in front of the X-ray source, after which we entered the control room, shut the lead door tight, and evaluated the X-ray characteristics of the sample by remotely activating the X-ray source.

## Data Availability

• All data reported in this paper will be shared by the lead contact upon request. • This paper does not report original code. • Any additional information required to reanalyze the data reported in this paper is available from the lead contact upon request.
