# Supplementary material for: Self-powered flexible all-perovskite X-ray detectors with high sensitivity and fast response
Source: iScience. 2021 Jul 30;24(8):102927. doi: 10.1016/j.isci.2021.102927 (PMC8365385; doi:10.1016/j.isci.2021.102927)
Supplement: Document S1. Figures S1–S4 and Table S1 [file mmc1.pdf]

iScience, Volume *24*

## Supplemental information

# Self-powered flexible all-perovskite X-ray detectors with high sensitivity and fast response

**Jin Hyuck Heo, Jin Kyoung Park, Yang (Michael) Yang, David Sunghwan Lee, and Sang Hyuk Im**

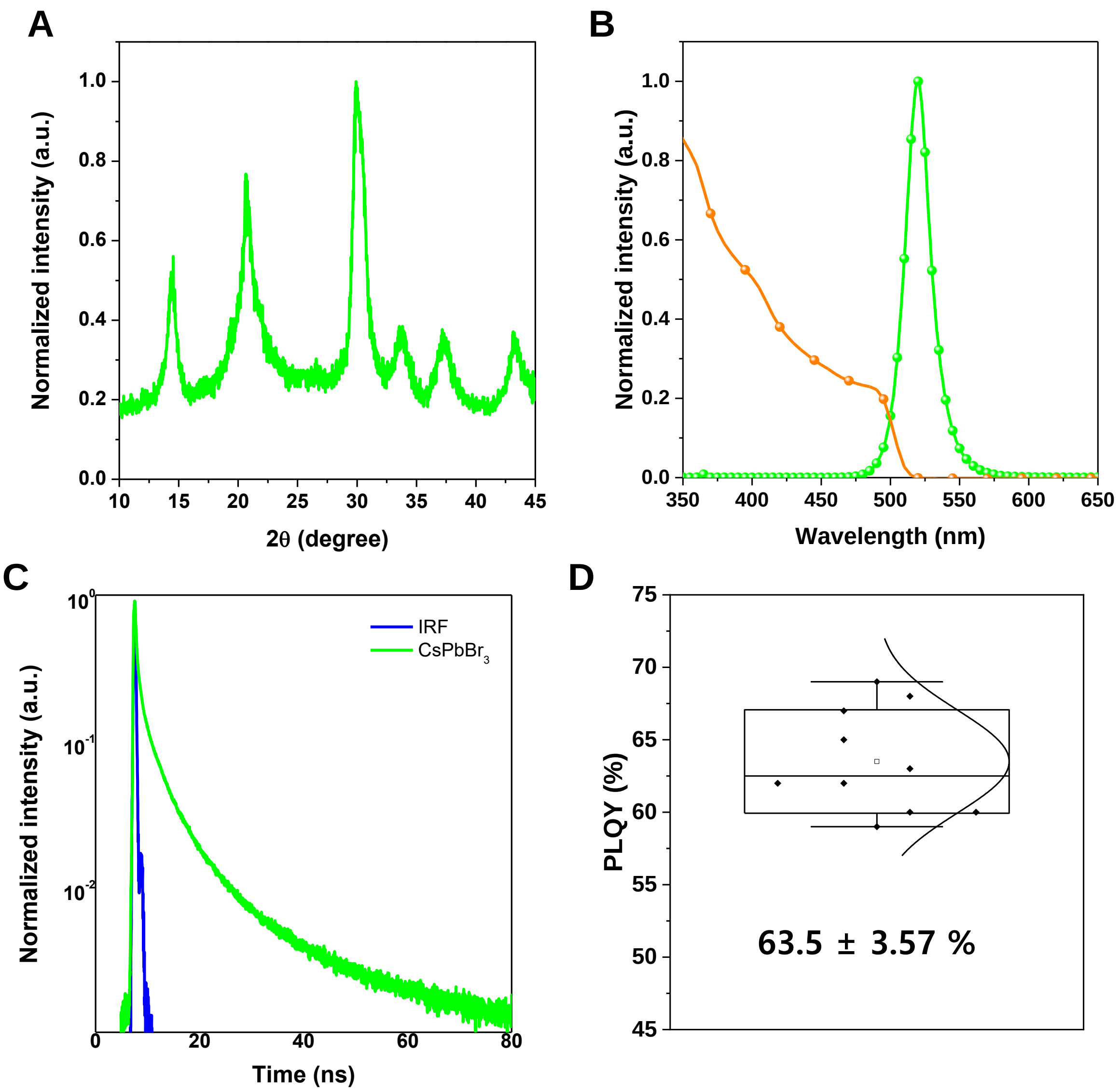


**Figure S1. Material and photoluminescent properties of $CsPbBr_3$ PNCs X-ray scintillator film, related to Figure 1.**

(A) X-ray diffraction (XRD) patterns of $CsPbBr_3$ PNCs.
(B) UV-visible and PL spectrum of $CsPbBr_3$ PNCs.
(C) transient PL decay curve of $CsPbBr_3$ PNCs X-ray scintillator film (excitation: 373 nm, emission: 520 nm).
(D) photoluminescence quantum yield (PLQY) of the scintillator film, represented in mean +/- standard deviation.

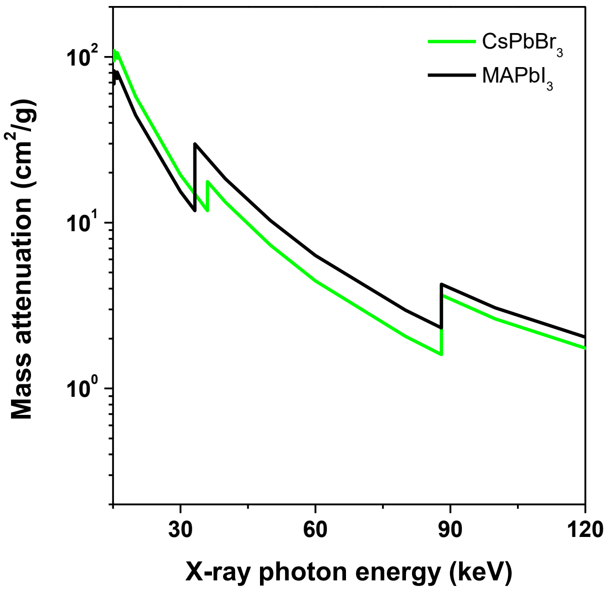


**Figure S2. Mass attenuation of $CsPbBr_3$ and $MAPbI_3$ perovskite with X-ray photon energy, related to Figure 3.**

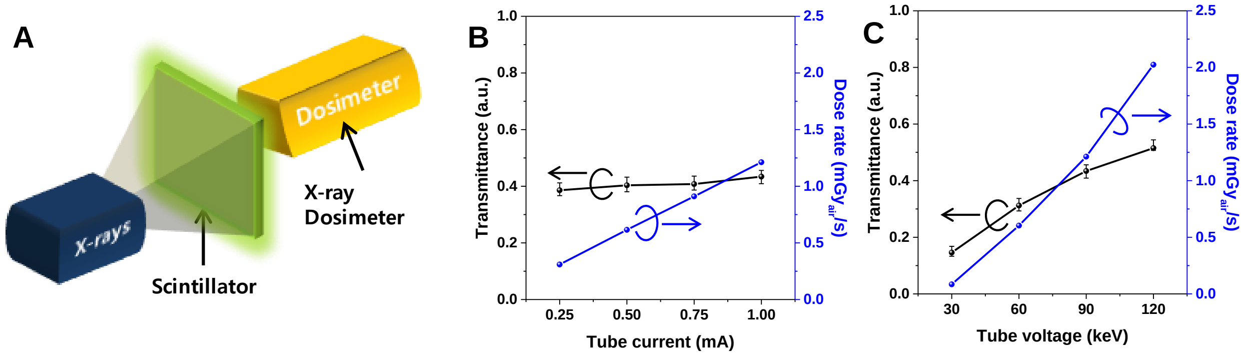


**Figure S3**. **X-ray detection exhibited by $CsPbBr_3$ PNCs scintillator film, related to Figure 3**.

(A) Schematic experimental set-up.
(B) tube current vs dose rate and tube current vs transmittance of $CsPbBr_3$ PNCs scintillator under fixed tube voltage of 90 keV.
(C) tube voltage vs dose rate and tube voltage vs transmittance under fixed tube current of 1 mA.
All data are represented as mean +/- standard deviation.

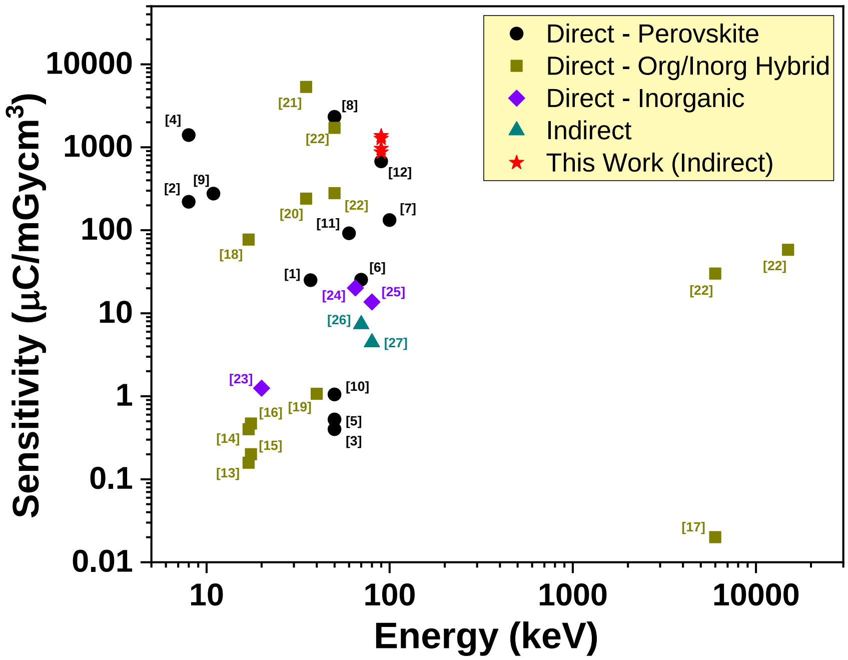


**Figure S4. Sensitivity comparison chart of various X-ray detectors, related to Figure 3 and Table S1.**

Black circles represent performance of direct-type perovskite X-ray detectors, dark yellow squares represent performance of direct-type organic/inorganic hybrid X-ray detectors, purple diamonds represent performance of direct-type inorganic X-ray detectors, green triangles represent performance of indirect-type X-ray detectors, and red stars represent performance of detectors in this work.

**Table S1**. **Performance comparison among recent state-of-the-art X-ray detectors and results from this work, related to Figure 3 and Figure S4.**

MA = methylammonium; BA = butylammonium; DMEDA = *N,N'*-dimethylethanediamine; F8T2 = poly([9,9-dioctylfluorenyl-2,7-diyl]-co-bithiophene); PTAA = poly(triarylamine); TIPS = triisopropylsilylethynyl; P3HT = poly(3-hexylthiophene); PFO = poly(9,9-dioctyfluorene); a-Se = amorphous-Se; GOS:Tb = terbium-doped gadolinium oxysulfide; CsI(Tl) = thallium-doped cesium iodide.
Related to **Figure 3** and **Figure S4**.

| Type | # | Year | Detecting Material | X-ray Energy (keV) | Sensitivity ($\mu C/mGy_{air}cm^3$) | Operating Voltage (V) | DOI |
|---|---|---|---|---|---|---|---|
| Direct (Perovskite) | 1 | 2015 | $MAPbI_3$ | 37 | 25 | 0 | 10.1038/NPHOTON.2015.82 |
| | 2 | 2016 | $MAPbI_3$ | 8 | 220 | -20 | 10.1038/NPHOTON.2016.139 |
| | 3 | 2016 | $MAPbBr_3$ | 50 | 0.4 | -0.1 | 10.1038/NPHOTON.2016.41 |
| | 4 | 2017 | $MaPbBr_3$ | 8 | 1400 | -7 | 10.1038/NPHOTON.2017.43 |
| | 5 | 2017 | $Cs_2AgBiBr_6$ | 50 | 0.525 | -50 | 10.1038/s41566-017-0012-4 |
| | 6 | 2017 | $MAPbI_3$ | 70 | 25.27 | -200 | 10.1038/NPHOTON.2017.94 |
| | 7 | 2017 | $MAPbI_3$ | 100 | 132.53 | -50 | 10.1038/nature24032 |
| | 8 | 2019 | $CsPbBr_3$ | 50 | 2320 | -1.2 | 10.1002/adma.201904405 |
| | 9 | 2020 | $(BA)_2(MA)_2Pb_3I_{10}$ | 10.91 | 276 | -1 | 10.1126/sciadv.aay0815 |
| | 10 | 2020 | $(DMEDA)BiI_5$ | 50 | 1.05 | -300 | 10.1039/C9TC06313G |
| | 11 | 2020 | $MAPbI_3$/Nylon | 60 | 91.83 | -12 | 10.1038/s41566-020-0678-x |
| | 12 | 2020 | $MAPbI_3$ | 90 | 675 | -100 | 10.1038/s41598-020-76647-5 |
| Direct (Org/Inorg hybrid) | 13 | 2009 | F8T2 | 17 | 0.1582 | -50 | 10.1117/12.829619 |
| | 14 | 2009 | PTAA | 17 | 0.4 | -300 | 10.1063/1.3225909 |
| | 15 | 2012 | PTAA-$Bi_2O_3$ | 17.5 | 0.2 | -200 | 10.1088/0957-4484/23/23/235502 |
| | 16 | 2013 | F8T2-$Bi_2O_3$ | 17.5 | 0.468 | -50 | 10.1088/0022-3727/46/27/275102 |
| | 17 | 2013 | F8T2 | 6000 | 0.02 | -150 | 10.1088/0031-9155/58/13/4471 |
| | 18 | 2016 | TIPS-pentacene | 17 | 77 | 0.2 | 10.1038/ncomms13063 |
| | 19 | 2016 | PbS-P3HT:PCBM | 40 | 1.068 | -30 | 10.1016/j.orgel.2016.03.023 |
| | 20 | 2017 | PFO-$Bi_2O_3$ | 35 | 240 | -80 | 10.1063/1.4986345 |
| | 21 | 2017 | TIPS-pentacene | 35 | 5333 | -3 | 10.1002/aelm.201600409 |
| | 22 | 2018 | P3HT:PCBM-$Bi_2O_3$ | 50 | 1712 | -10 | 10.1038/s41467-018-05301-6 |
| | 22 | 2018 | P3HT:PCBM-$Bi_2O_3$ | 50 | 280 | -10 | 10.1038/s41467-018-05301-6 |
| | 22 | 2018 | P3HT:PCBM-$Bi_2O_3$ | 6000 | 30 | -10 | 10.1038/s41467-018-05301-6 |
| | 22 | 2018 | P3HT:PCBM-$Bi_2O_3$ | 15000 | 58 | -10 | 10.1038/s41467-018-05301-6 |
| Direct (Inorganic) | 23 | 2000 | a-Se | 20 | 1.25 | -2000 | 10.1088/0022-3727/33/21/326 |
| | 24 | 2008 | CdZnTe:Cl | 65 | 20.2 | 290 | 10.1016/j.nima.2008.03.057 |
| | 25 | 2014 | CdTe | 80 | 13.68 | -250 | 10.1088/1748-0221/9/01/P01010 |
| Indirect | 26 | 2015 | GOS:Tb/P3HT:PCBM [a] | 70 | 7.35 | -10 | 10.1038/NPHOTON.2015.216 |
| | 27 | 2020 | CsI(Tl)/$MAPbI_3$ [a] | 80 | 4.46 | 0.6 | 10.3390/s20236872 |
| | | 2021 | $CsPbBr_3$/$MAPbI_3$ [a] | 90 | 960 [c], 870 [d] | 0 | This Work |
| | | 2021 | $CsPbBr_3$/$MAPbI_3$ [a] | 1 mA [b] | 1370 [c], 1270 [d] | 0 | This Work |

[a] Detecting materials are denoted as "X-ray scintillator/photodetector".
[b] Incident X-ray was obtained by maintaining constant tube current at 1 mA instead of tube voltage.
[c] Sensitivity value for rigid devices.
[d] Sensitivity value for flexible devices.
